# Effect of Microwave Irradiation at Different Stages of Manufacturing Unsaturated Polyester Nanocomposite

**DOI:** 10.3390/polym14214594

**Published:** 2022-10-29

**Authors:** Andrey Shcherbakov, Anton Mostovoy, Amirbek Bekeshev, Igor Burmistrov, Sergey Arzamastsev, Marina Lopukhova

**Affiliations:** 1 Laboratory of Support and Maintenance of the Educational Process, Yuri Gagarin State Technical University of Saratov, Polytechnichskaya St., 77, 410054 Saratov, Russia; 2 Laboratory of Modern Methods of Research of Functional Materials and Systems, Yuri Gagarin State Technical University of Saratov, Polytechnichskaya St., 77, 410054 Saratov, Russia; 3 Laboratory of Polymer Composites, K. Zhubanov Aktobe Regional State University, Aliya Moldagulova Avenue 34, Aktobe 030000, Kazakhstan; 4 Engineering Center, Plekhanov Russian University of Economics, 36 Stremyanny Lane, 117997 Moscow, Russia; 5 Department of Ecology and Technosphere Safety, Yuri Gagarin State Technical University of Saratov, Polytechnichskaya St., 77, 410054 Saratov, Russia; 6 Department of Economics and Humanitarian Sciences, Yuri Gagarin State Technical University of Saratov, Polytechnichskaya St., 77, 410054 Saratov, Russia; 7 Department of Functional Nanosystems and High-Temperature Materials, National University of Science and Technology MISiS, Leninskiy Prospekt 4, 119049 Moscow, Russia

**Keywords:** polyester resin, carbon nanotubes, physical modification, homogenization, microwave treatment

## Abstract

The possibility of using microwave radiation at various stages of obtaining an unsaturated polyester composite modified with carbon nanotubes was studied. The optimal content of MWCNTs in the system was experimentally selected, having the best effect on the strength of the composite. The effect of the microwave field on the properties of a polyester composite during the microwave treatment of an oligomer, a polymerized composite, and MWCNTs before their addition into the oligomer was studied. The processes of the structure formation, the structure of the composite, the effect of the microwave radiation on MWCNTs, and the thermal stability of the resulting composites were considered.

## 1. Introduction

Due to their properties, polymer composite materials (PCMs) are used in many branches of industry and national economy. The basis of any PCM is a binder. Composites based on unsaturated polyester resin (UPR) are widely used due to relatively high physical and mechanical properties, ease of operation, and corrosion resistance—all this is combined with low cost and availability [1,2,3].

The properties of PCMs largely depend on the filler. Currently, the main direction of research in the field of creating new composites is the modification of polymer matrices with nanosized particles [4,5,6]. Nanosized particles have an abnormally developed surface with functional groups, which allows them to be incorporated into the material structure with the formation of chemical bonds [7]. There are a large number of studies on the modification of carbon nanotube (CNT) binders [8,9,10], graphene oxide (GO), metal oxides, halloysite nanotube (HNTs), and biodegradable fillers, among others. One of the most important things when using nanosized particles is their uniform distribution in the matrix. In previous research [11,12,13], the possibility of adding CNTs into UPR using solvents at the stage of homogenization was studied. Styrene, ethanol, methanol, and acetone were used as solvents. The best physico-mechanical characteristics of the composite were obtained by adding CNTs into styrene; the addition of nanoparticles into other studied solvents reduced the strength of the composite. Homogenization of CNTs by ultrasonic treatment of the binder is widespread; the effectiveness of this method has been confirmed in a number of works [11,14,15].

Particular attention is paid to the surface modification of nanosized particles for chemical interaction with the binder. Fatty acids, silanes, amines, sulfates, etc., are used as surfactants. Chen et al. [16] successfully modified UPR with hydrophobic nanosized zinc oxide (ZnO) particles. The surface treatment of ZnO was carried out using aminopropyltriethoxysilane and oleic acid, and the resulting particles were successfully integrated into the composite structure with the formation of chemical bonds. By adding three parts by mass of treated ZnO, the thermal stability of the resulting polymer increased when the temperature reached 365 °C, and the tensile and bending strengths of a relatively pure composite increased by 91% and 71%, respectively.

In [17], multi-walled carbon nanotubes (MWCNTs) were used as a filler; the authors described a possible mechanism for the chemical interaction of a filler with a polyester matrix, wherein an increase in strength and modulus in bending by 37% and 57%, respectively, were presented. The thermal stability of the composite improved, and the glass transition and melting temperatures increased by 6 °C and 10 °C, which was shown by thermogravimetric analysis (TGA) and differential scanning calorimetry (DSC) methods. Gao et al. [18] added surface-modified 1-N-butyl-3-methylimidazolium hexafluorophosphate and 3-aminopropyltriethoxysilane GO into UPR to improve the fire resistance of the composite combined with ammonium polyphosphate. TGA analysis showed a significant increase in the temperature of the beginning of degradation, the maximum temperature of degradation, and an increase in the proportion of residual carbon. The oxygen index increased from 24% for the initial polymer to 28.2% for the composition with modified GO, which enabled the achievement of the level V-0 by UL-94. The study of the combustion surface of the composite modified with GO using a scanning electron microscope (SEM) showed the formation of a smooth, even and dense surface relative to the composite modified only with ammonium polyphosphate, which prevents the transfer of heat, oxygen, and combustible gas from the combustion surface.

Several basic methods of physical modification of polymers are known, including ultrasonic treatment, high-frequency currents, ultraviolet, and laser processing, among others. Microwave radiation is worth noting, since the nature of its effect on thermosets has been poorly studied thus far, while there are virtually no works on the effect of microwave radiation on UPR. In [19], the possibility of modifying an epoxy composite using microwave radiation at the oligomer stage was studied. Optimal power and time of treatment resulted in an increase in the flexural and tensile strengths of the composite by 26% and 29%, respectively. A change in the structure of the composite was noted in the SEM photographs; microwave treatment led to an increase in the proportion of plastic fracture, which the authors relate to the formation of a denser three-dimensional cross-linked structure around the filler particles.

This article focuses on the effect of the addition of MWCNTs and microwave modification on the mechanical properties, curing process, thermal stability, structure, and physicochemical properties of the obtained polyester composite. The novelty of this work lies in the fact that the effect of the microwave field on the properties of a polyester composite during the microwave treatment of an oligomer, a polymerized composite, and MWCNTs before their addition into the oligomer was studied. The influence of microwave irradiation, applied at various stages of formation, on the processes of structure formation and the structure of polyester composites, ensuring the strengthening of polymer composites, was proven.

## 2. Materials and Methods

### 2.1. Objects

**Figure 1:**
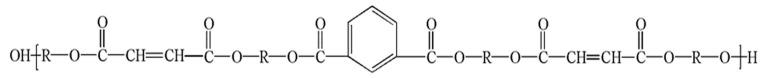
Unsaturated polyester oligomer.

Unsaturated polyester resin Aropol M105TB based on orthophthalic acid and curing initiator BUTANOX M-50 (methyl ethyl ketone peroxide) manufactured by Ashland Inc. was used to form the polymer matrix. Chemical structures of unsaturated polyester resin is shown in Figure 1.

**Figure 2:**
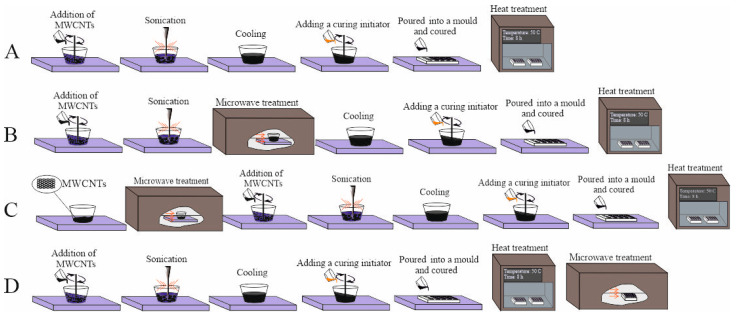
Schematic illustration for the preparation process of MWCNTs/UP nanocomposites: (**A**) UPR + MWCNTs; (**B**) UPR + MWCNTs + oligomer microwave treatment; (**C**) UPR + MWCNTs microwave treatment; (**D**) UPR + MWCNTs + composite microwave treatment.

To modify the polymer matrix, we used Taunit MWCNTs manufactured by NanoTechCenter Ltd. (Tambov, Russia), according to the specification; their specific surface area was ≥ 160 m^2^/g, bulk density 0.3–0.6 g/cm^3^, inner diameter 10–20 nm, and outer diameter 20–50 nm, with a length of at least 2 µm. In one of the ways to obtain composites, the MWCNTs were treated with microwave radiation before being added into the matrix (Figure 2C). In order to activate the functional groups on the surface and better distribute MWCNTs in the binder (Figure 2C), the treatment was carried out for 15 s at a frequency of 2.45 GHz with a radiated power of 1.3 kW.

### 2.2. Polyester Composite Fabrication Method

As a modifying additive, 0.025–0.1 parts by mass of MWCNTs were added into the unsaturated polyester resin by mechanical mixing for 10 min. Several ways of obtaining composites were proposed (Figure 2): (a) UPR + MWCNTs; (b) UPR + MWCNTs + oligomer microwave treatment; (c) UPR + MWCNTs microwave treatment; (d) UPR + MWCNTs + composite microwave treatment. MWCNTs tend to form agglomerates; to reduce their number and better distribute nanoparticles in the binder, ultrasonic treatment by the UZDN-2T device was used at a frequency of 22 ± 2 kHz with periods of 2 min for 15 min. To avoid overheating of the binder, the walls of the vessel in which ultrasonic treatment was carried out were cooled by running water. The curing process passes two main stages: curing at 25 °C for 24 h and final curing of the obtained composites at 50 °C for 8 h, after which the composite is kept for at least 24 h for gradual cooling and a relaxation processes.

### 2.3. Methods for Studying the Obtained Composites

The surface morphology of the samples was studied using a Tescan VEGA 3 SBH scanning electron microscope (Brno, Czech Republic). A DXR Raman Microscope (Thermo Scientific, Waltham, MA, USA) was used to measure Raman spectra. Tensile and flexural stresses and their respective elastic moduli were obtained using a WDW-5E Universal Electromechanical Testing Machine (Time Group Inc., Beijing, China) in accordance with ISO 527:12 and ISO 178:2019. Charpy impact strength was obtained using an LCT-50D pendulum impact tester (Beijing United Test Co., Beijing, China) according to ISO 179-1:2010. A DTAS-1300 thermal analyzer (Samara, Russia) was used to carry out differential scanning calorimetry (DSC) of polyester compositions under the following conditions: sample weight was 20 mg, heating interval was up to 400 °C, heating rate was 8 degrees per min^−1^; heat was determined by the heat flux (the derivative of heat with respect to time). The heat flux was determined by the temperature difference at two points of the measuring system at the same time. The mass of internodal polymer chains was determined by the ratio $M_{C}=\frac{3\mathrm{RT}\rho }{E_{P}}$, where R is the universal gas constant, T is the ambient temperature (K), ρ is the density of the composite (kg m^−1^), and E_P_ is the tensile modulus (Pa). IR spectra of the polymer were obtained using a Shimadzu IRTracer-100 (Tokyo, Japan). The change in mass, the rate of mass change, and the magnitude of thermal effects during heating of the samples were studied by thermogravimetric analysis using a MOM Q-1500 D Paulik-Paulik-Erdey derivatograph (Budapest, Hungary) under the following experimental conditions: sample mass—100 mg, medium—air, heating interval—25–1000 °C, heating rate—10 °C/min, relative error not exceeding 1%. Determination of heat resistance according to Vicat was carried out according to ISO 306:2004, method B50-load 50 N; the temperature rise rate was 50 °C/h.

## 3. Results and Discussion

### 3.1. Raman Spectroscopy of MWCNT Samples

**Figure 3:**
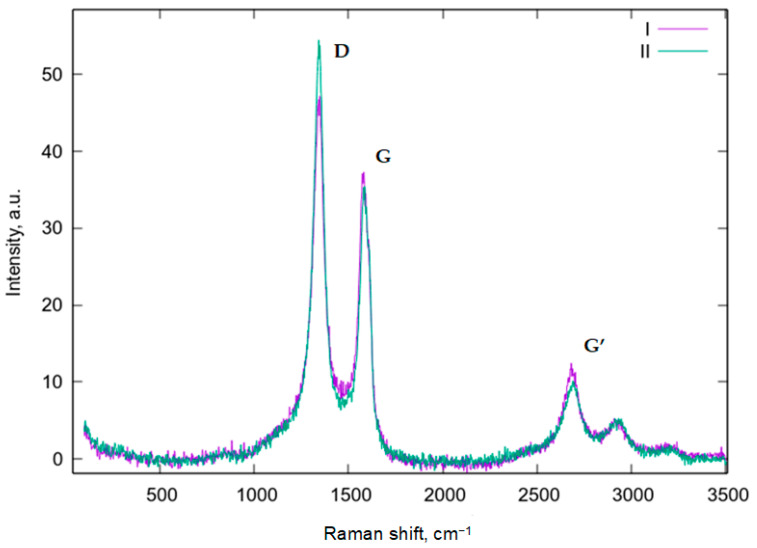
Raman spectra of initial (I) and microwave-treated (II) MWCNTs.

The effect of microwave treatment on the structure and the degree of imperfection of MWCNTs was studied by Raman spectroscopy. An analysis of the Raman spectra (Figure 3) of the initial (I) and microwave-modified (II) MWCNTs shows that they had the same set of lines corresponding to MWCNTs: D (1343 cm^−1^), G (1580 cm^−1^), and G’ (2680–2800 cm^−1^); however, the R value (ratio of peak D and peak G) for (I) was 1.268, which was significantly less than for (II), which was 1.538. Moreover, a small difference can be noted in the first line of the G’ band, which implies a more regular structure of carbon (I) atoms, and therefore, we can conclude that the microwave modification increased the number of defects on MWCNTs (Figure 3) [20,21,22].

### 3.2. Selection of the Optimal Amount of MWCNTs in a Polyester Composition

To obtain a composite with the highest strength characteristics and its further microwave modification, it is necessary to determine the optimal content of MWCNTs in the polymer matrix. The optimal amount of MWCNTs was selected on the basis of the physical and mechanical characteristics of polyester composites with a MWCNT content of 0.025 to 0.1 parts by mass (Table 1). The addition of 0.05 parts by mass of MWCNT made it possible to obtain the highest bending stress + 27.3%, flexural modulus + 18.7%, tensile strength + 31%, and impact strength + 58%. The addition of MWCNTs resulted in the decrease in the tensile modulus by ≈10%.

### 3.3. Influence of Microwave Irradiation on the Mechanical Properties of Polyester Composites

To improve the deformation–strength properties of polyester composites, microwave modification was used at various stages of composite fabrication. Microwave modification of the oligomer after the addition of MWCNT provides an increase in the impact strength of the polyester composite by 50%, with other physical and mechanical characteristics being maintained (Table 2). The microwave treatment of the finished composite made it possible to increase the tensile stress and impact strength of the composite by 18 and 23%, respectively. Direct microwave modification of MWCNTs, before their addition into the polyester composition, turned out to be more effective. A polyester composite containing microwave-modified MWCNTs was characterized by higher strength properties: flexural stress, tensile stress, and tensile modulus increased by 11%, 12%, and 21%, respectively, as compared to a polyester polymer containing unmodified MWCNTs (Table 2).

### 3.4. Study of the Structure Polyester Composite

**Figure 4:**
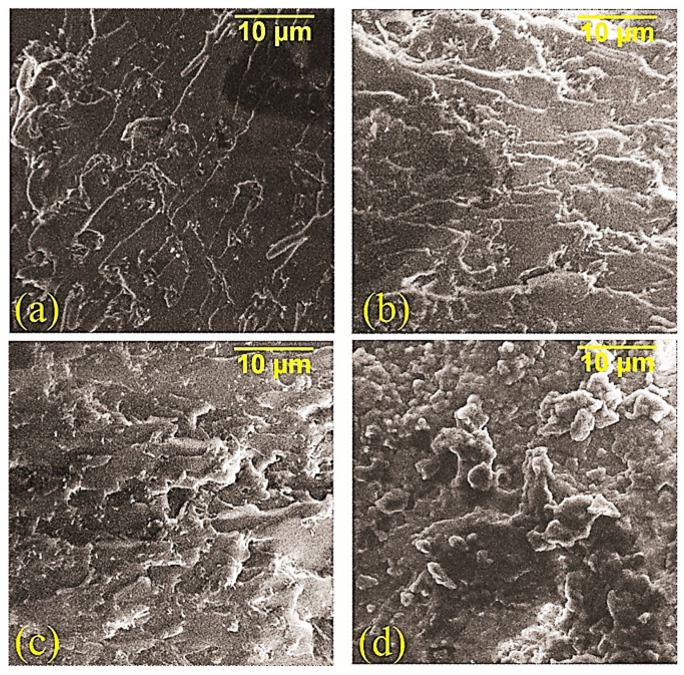
SEM data of composites: (**a**) PC; (**b**) PC + MWCNT; (**c**) PC + MWCNT + microwave (oligomer); (**d**) PC + MWCNT + microwave (composite).

**Figure 5:**
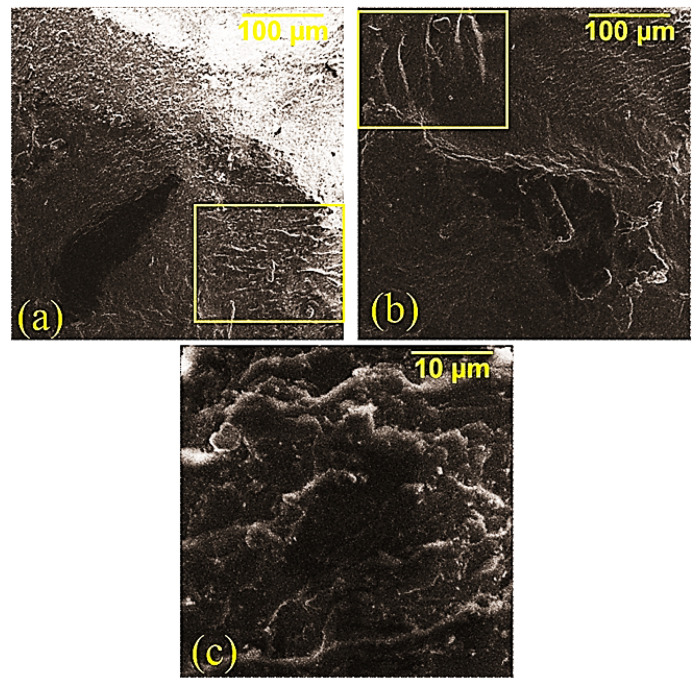
SEM data of a polyester composite modified with microwave-treated MWCNTs: (**a**) 500×: (**b**) 500×: (**c**) 5000×.

The surface structure of the composites, as well as the effect of adding MWCNTs into the composition and microwave modifications, were studied by SEM. The initial composite (Figure 4a) is characterized by sharp edges with a relatively smooth surface. In [19,23,24], it is noted that a similar surface structure is observed in composites with a weak ability to resist the formation of microcracks during degradation. The addition of MWCNTs into the polyester matrix (Figure 4b) makes the structure of the composite more relief, which can be seen in the formation of deepenings on the surface, which confirms the increase in the ability of the composite to resist the formation of microcracks when MWCNTs are added into the composition. The microwave treatment of the composition with MWCNTs at the oligomer stage led (Figure 4c) to the formation of a “wavy” surface structure, and the microwave treatment of the cured composite filled with MWCNTs led to significant changes in the structure, thus making the destruction softer. This destruction was accompanied by stretching matrix (Figure 4d), which allowed for a further increase in the strength of the composite. The effect of the microwave radiation on the cured composite can be explained by relaxation processes triggered by microwave radiation due to the direct effect on the three-dimensional cross-linked structure of the polymer. The treatment of MWCNTs in the microwave field before their addition into the oligomer significantly affected the structure of the composite, while a change in the nature of the degradation to partially viscous-flowing was observed, which was confirmed by the formation of strands on the surface of the composite (Figure 5).

### 3.5. Differential Scanning Calorimetry of Polyester Compositions

For a correct and complete description of the effect of MWCNTs and microwave radiation on the properties of the resulting composite, it is necessary to study the processes of the structure formation during curing. The effect of adding MWCNTs into the polyester composition and microwave modification on the processes of the structure formation during the curing of an unsaturated polyester oligomer was studied by differential scanning calorimetry (DSC).

**Figure 6:**
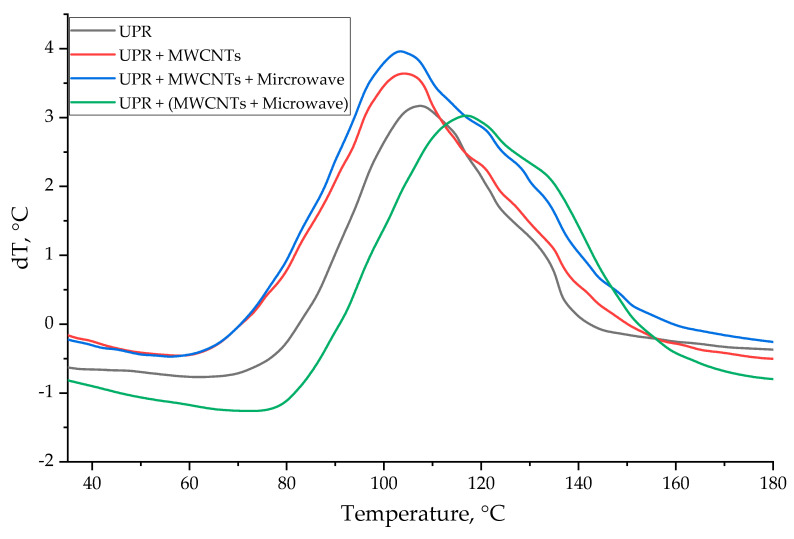
DSC data of polyester compositions.

The analysis of the DSC curves (Figure 6) allows for the assumption that both the addition of MWCNTs and the microwave modification have an effect on the processes of the structure formation. The curing process of an unsaturated polyester composite is divided into two main stages. At the first stage, the reaction is initiated, and the inhibitor contained in the oligomer is consumed (at dT < 0, Figure 6), and at the second stage, the composition is directly cured (at dT > 0, Figure 6). Blanco and colleagues speculate that the shoulder that appears on the graph in the region of 140–150 °C is the result of homopolymerization of the polyester oligomer as a result of providing the system with sufficient energy [25,26].

As a result of the addition of MWCNTs, the curing start temperature decreased from 81 to 69 °C, and the reaction enthalpy increased from 431 to 545 J/g (Table 3). MWCNTs are assumed to initiate the curing process and serve as additional cross-linking centers, which was confirmed by an increase in the degree of curing of the composite. Microwave treatment of an oligomer containing MWCNTs led to an increase in heat release from 545 to 647 J/g due to an increase in the intensity of the curing process and a shift in the final temperature of the reaction from 149 to 162 °C (Table 3). The process of the structure formation under even more severe conditions probably did not allow for the achievement of a significant increase in strength due to the formation of overstresses in the matrix. Microwave treatment of MWCNTs and their subsequent addition into the matrix, on the contrary, significantly increased the temperature at the start of the reaction from 69 to 91 °C and the temperature of the maximum heat release from 103 to 115 °C, while a decrease in the reaction enthalpy from 545 to 403 J/g was observed (Table 3). According to [27,28], the increase in the start temperature of the reaction and the decrease in enthalpy during the curing process was associated with the ability of MWCNTs to retain radicals due to their high specific surface area, as a result of which the MWCNT-styrene radical structure formed, and it was that structure that reacted with the polyester oligomer. Softer curing conditions were likely to make it possible to reduce the number of defects in the matrix and achieve high physical and mechanical characteristics.

### 3.6. IR Spectroscopy of Composites

**Figure 7:**
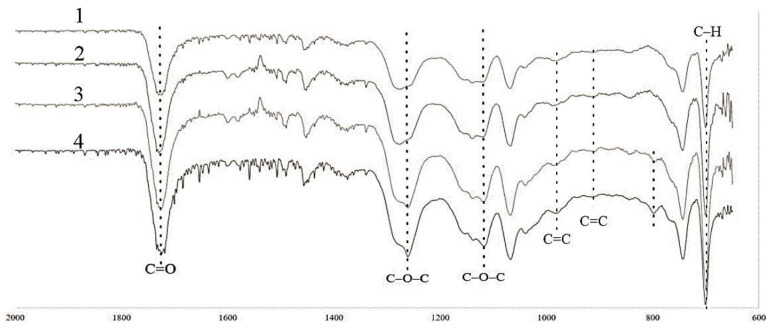
IR spectroscopy of samples: 1—neat UPR; 2—UPR + MWCNTs; 3—UPR + MWCNTs + oligomer microwave treatment; 4—UPR + microwave treatment MWCNTs.

The interaction of the polyester oligomer with MWCNTs and the effect of microwave modification were studied by IR-Fourier spectroscopy in the range from 2000 to 600 cm^−1^ (Figure 7). The choice of this particular range was due to the presence of the main peaks characterizing the polyester composite. The spectrum of the unsaturated polyester composite was used as a reference.

The main peaks of the polyester composite included an intense peak in the range of 1725 cm^−1^ > C=O of the carboxyl group, and 1259 cm^−1^ and 1120 cm^−1^ symmetrical C–O–C groups. The peaks in the range of 1145 cm^−1^ and 1075 cm^−1^ were characterized by C–O stretching vibrations. It was necessary to separately mark the residual peaks at 982 cm^−1^ and 912 cm^−1^ related to the unreacted C=C groups of polyester and styrene, respectively [10,11,29,30]. The peak in the range of 700 cm^−1^ was associated with vibrations of the C–H bond, the intensity of which depended on the degree of composite curing. The addition of MWCNTs into the polyester matrix had virtually no effect on the spectral pattern. Although there were carboxyl groups on the MWCNTs surface, which can react during curing, it was impossible to assess the actual degree of interaction due to the small amount of the filler in the system. The microwave effect of the oligomer with MWCNTs preliminarily added into it probably made it possible to activate the functional groups on the MWCNT surface, which resulted in an increase in the intensity of the C–O–C peaks, as well as in the appearance of a new peak in the range of 800 cm^–1^. Microwave treatment of MWCNTs immediately prior to their addition into the oligomer led to an additional increase in the intensity of the peaks at 1259, 1120, and 800 cm^–1^, which proved the activation of functional groups on the MWCNT surface due to microwave modification and their interaction with the functional groups of the polyester composition.

### 3.7. Thermogravimetric Analysis of Polyester Composites

**Figure 8:**
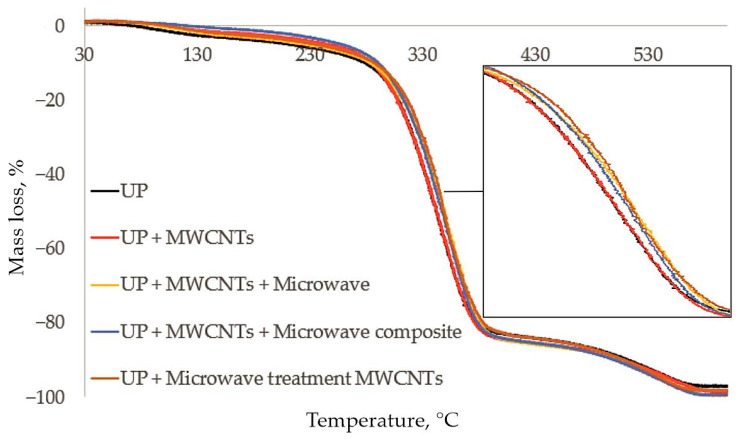
Data of thermogravimetric analysis polyester samples.

The thermal stability and behavior of the composites during thermal degradation was studied by the TGA method. Figure 8 shows the curves of thermogravimetric analysis for polyester composites. The results of TG and heat resistance are presented in Table 4.

A weight loss of 1% was taken as the initial temperature of composite degradation. The process of thermolysis of an unsaturated polyester composite includes three stages. At the first stage, unreacted styrene volatilizes, and water dehydrates at temperatures of 100–280 °C. The second stage of the composite decomposition in the temperature range of 280–390 °C involves splitting of the three-dimensional cross-linked structure of the composite until its fragments become small enough to volatilize. At the final stage of the decomposition at temperatures up to 580 °C, the composite decomposes to the formation of carbonized structures consisting only of the carbon skeleton of the composite. As can be seen from Figure 8, the addition of MWCNTs did not worsen the thermal stability of the composite; moreover, the temperature of the beginning of the main degradation stage increased by 28 °C (Table 4). Microwave modification of the polyester nanocomposite made it possible to increase the heat resistance without changing the thermal stability of the composite. The microwave treatment of the finished composite had a strong effect on the temperature of the start of degradation, which is probably associated with the post-curing of the composite during treatment and, as a result, a decrease in the yield of unreacted styrene at the initial stage. An increase in the degradation temperature at temperatures of 280–390 °C was noted, showing an increase in the thermal stability of the composite. The addition of microwave-treated MWCNTs into the polyester composition results in the decrease in the thermal stability of the composite at thermolysis temperatures in comparison with the initial composite, which indirectly confirms the change in the three-dimensional cross-linked structure of the composite during curing.

### 3.8. Comparison of Developed Composites with Analogues

The developed composites have physico-mechanical characteristics comparable to those of the existing analogs and often cases exceeding them, which confirms the scientific novelty and significance of the results obtained (Table 5).

## 4. Conclusions

The addition of MWCNTs and microwave modification of the polyester matrix affects the strength indices, processes of the structure formation, morphology, and thermal stability of the composite. The addition of MWCNTs into the polyester matrix increased the strength of the composite, which is probably associated with a change in the structure of the composite as a result of the chemical interaction of functional groups on the MWCNT surface with the oligomer, which is confirmed by DSC data. Microwave treatment of the polyester matrix at the oligomer stage initiated the curing reaction; at the same time, a change in the nature of the destruction of the sample with the formation of deepenings was observed, indicating the ability of the composite to resist the formation of microcracks. The treatment of the polymerized composite in the microwave field changed its structure, due to relaxation processes, increasing the strength indices. The use of MWCNTs pre-treated in the microwave field had a significant effect on the processes of the structure formation, while the reaction rate decreased due to the trapping of styrene radicals on the surface of MWCNTs as a result of activation of the surface of carbon particles in the microwave field. The change in the nature of the reaction was observed in the appearance of a new peak in the IR spectra in the region of 800 cm^−1^ and an increase in the intensity of the peaks at 1259, 1120 cm^−1^; a similar picture with a lower peak intensity was noted during microwave treatment of polyester at the oligomer stage. The improvement of the thermal stability of composites was noted upon the addition of MWCNTs and microwave modification at the stage of the oligomer and composite.

## Figures and Tables

**Table 1:** Selection of the optimal amount of MWCNTs in a polyester composition.

The Content of MWCNTs in the Polyester Composite, Parts by Mass	σ_ben_, MPa	E_ben_, MPa	σ_ten_, MPa	E_ten_, MPa	a_im_, kJ/m^2^
0	66 ± 3.3	2245 ± 112	32 ± 1.6	1330 ± 66	2.8 ± 0.14
0.025	79 ± 3.9	2520 ± 126	36 ± 1.8	1200 ± 60	4.2 ± 0.21
0.05	85 ± 4.2	2665 ± 133	42 ± 2.1	1220 ± 61	4.5 ± 0.22
0.075	78 ± 3.9	2620 ± 131	40 ± 2.0	1200 ± 60	4.6 ± 0.23
0.1	68 ± 3.4	2085 ± 104	35 ± 1.7	1190 ± 59	4.3 ± 0.21

Note: σ_ben_—bending stress; E_ben_—modulus of elasticity in bending; σ_ten_—tensile strength; E_ten_—tensile modulus of elasticity; a_im_—impact strength.

**Table 2:** Properties of polyester composites.

Stage of Microwave Treatment of UPR + 0.05 MWCNT Composite	σ_ben,_ MPa	E_ben_, MPa	σ_ten_, MPa	E_ten_, MPa	a_im_, kJ/m^2^	M_c_, kg/mol
-	85 ± 4.2	2660 ± 133	42 ± 2.1	1220 ± 61	4.5 ± 0.22	7.24
Oligomer	82 ± 4.1	2860 ± 143	43 ± 2.1	1120 ± 56	6.7 ± 0.33	7.53
Composite	89 ± 4.4	2840 ± 142	50 ± 2.5	1270 ± 63	5.5 ± 0.27	7.38
MWCNT	94 ± 4.7	2620 ± 131	47 ± 2.3	1480 ± 74	2.5 ± 0.12	5.84

Note: σ_ben_—bending stress; E_ben_—modulus of elasticity in bending; σ_ten_—tensile strength; E_ten_—tensile modulus of elasticity; a_im_—impact strength; M_c_—molecular weight of internodal chains.

**Table 3:** Results of differential scanning calorimetry of polyester compositions.

Polymer/Stage of Microwave Treatment	T_start_–T_end_ T_max_, °C	T_glass_, °C	H, J/g
UP/-	81–152 108	93	431
UP + 0.05MWCNT/-	69–149 102	84	546
UP + 0.05MWCNT/Oligomer	69–162 102	85	647
UP + 0.05MWCNT/MWCNT	91–152 115	102	403

Note: Tstart, Tend—temperature of the start and end of the curing process; Tmax—the temperature of the maximum heat release during curing; H—thermal effect of the reaction.

**Table 4:** Thermogravimetric analysis results.

Polymer/Stage of Microwave Treatment	Vicat Heat Resistance, °C	Thermolysis Temperature, °C, at Weight Loss of Polyester Composite, %										
		1	10	20	30	40	50	60	70	80	90	97
UP/-	140	94	279	308	322	332	342	351	361	378	511	578
UP + 0.05MWCNT/-	168	122	284	308	322	332	343	353	362	376	506	566
UP + 0.05MWCNT/oligomer	176	114	283	312	327	339	349	358	367	380	501	555
UP + 0.05MWCNT/composite	167	168	290	313	327	338	346	356	365	378	499	552
UP + 0.05MWCNT/MWCNTs	140	122	288	316	330	341	349	357	368	383	507	556

**Table 5:** Comparison of developed composites with analogues.

Composition	σ_ben,_ MPa	E_ben_, MPa	σ_ten_, MPa	E_ten_, MPa	a_im_, kJ/m^2^
UPR + MWCNTs + microwave	89 ± 4.4	2840 ± 142	50 ± 2.5	1270 ± 63	5.5 ± 0.27
Analogs					
UPR + oil palm shell + MWCNTs [13]	60 ± 3.0	4150 ± 207	38 ± 1.9	-	-
UPR + OH functionalized MWCNTs [31]	48 ± 2.4	2787 ± 139	26 ± 1.3	1250 ± 62	2.3 ± 0.11
UPR + MWCNT (suspension in tetrahydrofuran) [10]	-	-	35 ± 1.7	1500 ± 75	4.6 ± 0.23
UPR + MWCNTs [17]	122 ± 6.1	8900 ± 445	40 ± 2.0	-	-
UPR + MWCNTs [32]	65 ± 3.2	3600 ± 180	27 ± 1.3	2390 ± 119	-

Note: σ_ben_—bending stress; E_ben_—modulus of elasticity in bending; σ_ten_—tensile strength; E_ten_—tensile modulus of elasticity; a_im_—impact strength.
